# Pathogenic variants of *BUB1* and *BUBR1* genes are not prioritized in screening tests of couples with aborted aneuploid fetuses 

**DOI:** 10.22099/mbrc.2024.51170.2037

**Published:** 2025

**Authors:** Raziyeh Gorji, Parnaz Borjian-Boroujeni, Masood Bazrgar

**Affiliations:** 1 Department of Molecular Genetics, Faculty of Basic Sciences and advanced Technologies in Biology, University of Science and Culture, ACECR, Tehran, Iran; 2 Department of Genetics, Reproductive Biomedicine Research Center, Royan Institute for Reproductive Biomedicine, ACECR, Tehran, Iran

**Keywords:** Aneuploidy, Spontaneous abortion, BUB1, BUBR1

## Abstract

Chromosome aberrations certainly aneuploidie are the cause of the majority of spontaneous abortions in humans. *BUB1* (budding uninhibited by benzimidazole 1) and *BUBR1* (BUB1 mitotic checkpoint serine/threonine kinase B) are two key proteins mediating spindle-checkpoint activation that play a role in the inhibition of the anaphase-promoting complex/ cyclosome (APC/C), delaying the onset of anaphase and ensuring proper chromosome segregation. This study aimed to evaluate the probable roles of *BUB1* and *BUBR1* pathogenic variants in abortion of the fetuses with aneuploidy. Fifty aborted fetuses with approved aneuploidy using array comparative genomic hybridization (aCGH) were included. *BUB1* and *BUBR1* genes were studied using the Sanger sequencing for the single nucleotide variant (SNV) detection, certainly rs121909055 and rs28989185 as the pathogenic target variants. The sequencing results were analyzed by finch TV software.Neither homozygous nor heterozygous variant of the targeted SNVs was observed in the samples. No other SNV was detectable in the analyzed parts of the *BUB1* and *BUBR1* genes in all samples. Since the allele frequencies of the variants of interest were zero in 50 studied samples, these SNVs would not be prioritized for screening in the parents with a history of miscarriage due to aneuploidy.

## INTRODUCTION

Approximately, 10-15% of clinically recognized pregnancies end to spontaneous abortion [1]. Fetal chromosomal abnormalities especially aneuploidy play an important role in spontaneous abortion, indeed, aneuploidy demonstrates a significant contribution of chromosomal abnormalities in abortion [2]. During the chromosome segregation in cell division, if an error occurred and the cell was not able to repair the problem, it would cause aneuploidy which can lead to birth defect, developmental disorders and cancer [3]. The incidence of these errors in embryonic development increases with maternal age [4]. One of the mechanisms of cell division fidelity is the spindle assembly checkpoint (SAC). During mitosis when the cycle is passing from metaphase to anaphase, SAC mechanism controls attachment of all chromosomes to spindle microtubules. In case that kinetochore is unattached, SAC activates and delays anaphase by inhibition of anaphase-promoting complex/cyclosome (APC/C) activation to correct the connection between sister chromatid and kinetochore [5]. At the beginning of SAC activation, a kinase known as MPS1 creates a binding site on Knl1, a component of kinetochore KMN network (Knl1, Mis12 and Ndc80), by its phosphorylation. Bub1-Bub3 complex attaches to Knl1 binding site. *Bub1 *attachment is essential for recruitment of other SAC components such as Mad1, Mad2, *BubR1* and Cdc20 to set up the mitotic checkpoint complex (MCC) [5].

*BUB1* (budding uninhibited by benzimidazoles-1) and *BUBR1* (BUB1 mitotic checkpoint serine/threonine kinase B) studies are mainly limited to analysis of these two genes in mouse embryos. Using tamoxifen to inactive *Bub1* gene in mouse embryos showed developmental arrest and premature centromere separation and also eventuated defective mitosis, gastrulation and organogenesis [6]. Knocking out of the *Bubr1* gene also results in aneuploidy and mosaic variegated aneuploidy (MVA) which leads to growth retardation or even embryonic death [7]. 

Regarding the critical role of *BUB1 *and* BUBR1* genes in chromosome segregation, this study investigated the potential contribution of their pathogenic variants (rs121909055 and rs28989185) to aneuploidy in aborted fetuses by Sanger sequencing for Single Nucleotide Variant (SNV) detection. These variants have been previously reported to disrupt the spindle assembly checkpoint mechanism, leading to chromosomal missegregation. The rs121909055, NM_004336.5(*BUB1*):c.1475C>A (p.Ser492Tyr), has been classified as Pathogenic in ClinVar. It is associated with increased cancer risk and contributes in chromosomal instability [8]. The rs28989185, NM_001211.6(*BUB1B*):c.3035T>C (p.Leu1012Pro), variant in the *BUBR1* gene is likely pathogenic. Based on the study done in the year 2010, biallelic mutations in this gene including this variant were shown to impair the Spindle Assembly Checkpoint (SAC), leading to aneuploidy, Mosaic Variegated Aneuploidy (MVA) syndrome, and cancer predisposition. If this variant causes functional changes in the *BUBR1* protein, it may follow a similar pathogenic mechanism as other mutations in this gene, supporting its pathogenicity [9]. The rs34998711, NM_001211.6(*BUB1B*):c.3011A>G (p.Asn1004Ser), in the *BUBR1* gene has been classified as likely benign based on submissions in the ClinVar database, which are supported by evaluations using established ACMG guidelines [10]. However, its location within the coding region of the *BUBR1* gene and its nature as a missense mutation affecting protein structure raise questions about its potential impact on Spindle Assembly Checkpoint (SAC) function under certain conditions. Interestingly, the rare rs34998711 variant lies in proximity to other pathogenic mutation of the *BUBR1* gene, the rs28989185. This raises the possibility that rs34998711 could exert subtle effects on protein interactions or expression levels, particularly in the context of compound heterozygosity or interaction with nearby variants.

## MATERIALS AND METHODS

This study was performed from January 2020 to May 2021. Array Comparative Genomic Hybridization (aCGH) was used to detect aneuploidy in the aborted fetuses. After labeling patient DNA and reference DNA with different fluorescent dyes and hybridizing them to microarray slides (Agilent), the slides was scanned by a laser scanner (InnoScan 710, Innopsys). Fifty fetal products of conception with confirmed aneuploidy via aCGH were collected from mothers aged <37 who had spontaneous miscarriages. All the couples signed an informed consent before sampling. Clinical history and pedigree of all couples referred to the center were recorded, and the inbreeding coefficient was calculated. Parameters studied included the type of aneuploidy, gestational week of abortion, maternal age, consanguinity, history of abortion, number of abortions, and sex. DNA was extracted by using salting out method. Samples with maternal DNA contamination were not included in the study. For maternal cell contamination testing, maternal blood was collected and analyzed by Short Tandem Repeat fingerprinting. Measuring of quality and concentration of the DNA was done by NanoDrop Spectrophotometer (NanoDrop Technologies, Inc.). 

Pathogenic variants of *BUB1* and *BUBR1* genes were selected using Clinvar and UCSC databases, https://www.ncbi.nlm.nih.gov/clinvar/, and https://genome.ucsc.edu/; below is the description of the pathogenic variants. We analyzed a pathogenic missense variant of *BUBR1*, rs28989185 (T>C), in this area which is a missense mutation . The rs34998711 on the exon 23 of the *BUBR1* gene, that leads to a missense mutation (A>G) nearby the rs28989185 also was analyzed. The rs121909055 was another variant of interest that located on the exon 13 of the *BUB1* gene, which changing of single nucleotides A or T to nucleotide G causes a missense mutation. According to the position of the variants, a pair of primers were designed, including the variants of interest, based on the human genome reference sequence. The designed primers for the *BUBR1* gene were 5'-TAGTTCTTCCCTGGGCTTTCAAA-3' as forward and 5'-AGTT GGCTACTCTGTCTCATCAC-3' as revers; and for the *BUB1* gene were 5'-ATAATCCAGAC CAACCACTCAATC-3' as forward and 5'-CCTCTACCAGTGAAGGCTCAA-3' as reverse. PCR reaction mixtures contained 0.5µl of each primer, 10µl of Taq DNA pol.master mix red (Amplicon), 1µl of DNA template and 18 µl  of dH_2_O. The reaction was performed with an initial denaturation at 94˚C for 4 min and 1 cycle followed by: denaturation at 94˚C for 30 seconds, annealing at 60˚C for 45 seconds, extension at 72˚C for 1 min, for 25 cycles, then final extension was carried out at 72˚C for 10 min. Due to the size of our PCR products, which were longer than 700bp, they were electrophoresed on 1% agarose gel. The results of sanger sequencing were analyzed using FINCH TV software and the sequences were aligned with the original sequences through the BLASTn (Nucleotide BLAST) website.

## RESULTS AND DISCUSSION

In the survey of the 50 studied cases, the most common types of aneuploidy, gender distribution of aborted fetuses, and the consanguinity of the couples is summarized in the Table 1. The occurrence of 78% of the abortions had been in the first trimester and 22% had occurred in the second trimester of pregnancy. Concerning the number of abortions, 28% of cases had no history of previous abortion, 26% experienced an abortion compared to 22% with two abortions, 2% with three and 6% had more than three abortions. The others did not have any record. After sequencing all samples related to pathogenic variants of interest and their analyses, it was found that all samples had normal genotypes in terms of variants in these regions and alignment with the reference sequences (Fig. S1). No genetic changes were observed in their sequenced upstream and downstream areas.

**Table 1:** Types of aneuploidy, consanguinity of couples, and gender distribution of aborted fetuses and their frequencies

**Type of aneuploidy**	**Subcategory**	**Frequency N(%)**
	Numerical abnormalities of chromosomes 13,18,21 and X	36 (72%)
	Trisomy 15	5 (10%)
	Trisomy 22	2 (4%)
	Other types of aneuploidies	7 (14%)
**Fetus gender**	Female	28 (56%)
	Male	16 (32%)
	Unknown	6 (12%)
**Couples degree of consanguinity**	First cousins	11 (22%)
	Second cousins	1 (2%)
	No consanguinity	34 (68%)
	Unknown	4 (8%)

Genetic defects, especially chromosomal abnormalities, account for about half of spontaneous abortions [11]. Molecular mechanisms, specifically those involved in chromosome separation during division, significantly influence aneuploidy. Spindle assembly checkpoint (SAC) prevents premature chromosome separation [5]. *BUB1*(MIM: 602452) and *BUBR1 *(MIM: 602860) are key proteins in the SAC that inhibit the anaphase-promoting complex/ cyclosome (APC/C) to ensure accurate chromosome segregation by delaying the transition of metaphase to anaphase [4]. Few studies have explored the association of these genes with abortion without considering aneuploidy, suggesting that reduced levels of *BUB1* and *BUBR1* proteins contribute to abortion [7, 12]. Furthermore, no studies have investigated these variants in abortion products, including aneuploid ones [13].

This study examined the roles of *BUB1* and *BUBR1* gene variants in fetal aneuploidy, hypothesizing that pathogenic variants might contribute to chromosomal missegregation and miscarriages. However, non of the investigated variants (rs121909055, rs34998711, and rs28989185) were identified in the 50 aneuploid aborted fetuses studied, suggesting these variants are not major contributors to aneuploidy in spontaneous abortion. This aligns with their rare occurrence in the general population, with allele frequencies of 0.000001859 for rs121909055 (*BUB1*), 0.0006034 for rs34998711, and 0.0001369 for rs28989185 (*BUBR1*) in the gnomAD database. Our findings suggest that other factors, such as gene alterations or environmental influences, may play a more significant role in aneuploidy-related miscarriages.

The findings of one study underscores the complexity of the molecular mechanisms underlying aneuploidy, suggesting that dysregulation of other components of the SAC, like MAD1, can also contribute to chromosomal missegregation [14]. This could be a complemen-tary to our study, which did not find specific *BUB1* and *BUBR1* variants but does not rule out the involvement of SAC-related proteins in aneuploid abortions. Moreover, another study highlights the importance of broad genetic investigations beyond targeted gene analysis. Their findings suggest that a wide array of genetic variations, potentially including non-coding regions and regulatory elements, might contribute to recurrent pregnancy loss [15]. Despite the absence of these specific variants in our study, the potential involvement of *BUB1* and *BUBR1* in aneuploidy cannot be entirely ruled out. It is possible that other less characterized variants or regulatory mutations in these genes or their interaction with other molecular pathways could contribute to chromosome missegregation. 

Aneuploidy-related mutations, though crucial in fertility issues like abortion, are rare [16], and the 50-sample size may not fully represent the population. A larger sample could increase the chances of discovering such mutations. Despite this, our study offers novel insights and lays the groundwork for future research, emphasizing the need for comprehensive genetic screening. While our targeted approach did not yield positive results, whole exome or genome sequencing may uncover other genetic factors. Integrating genomic data with environmental and clinical factors could provide a more complete understanding.

 In conclusion, while *BUB1 *and *BUBR1* are essential for chromosome segregation, the variants studied here are unlikely major contributors to aneuploidy in spontaneous abortions. Our findings, along with others, highlight the complexity of the genetic basis of aneuploidy-related miscarriages and the need for broader investigations. Further research is needed to explore the multifactorial nature of pregnancy outcomes.
