# Supplementary material for: Pathogenic variants of BUB1 and BUBR1 genes are not prioritized in screening tests of couples with aborted aneuploid fetuses
Source: Mol Biol Res Commun. 2025;14(2):143–7. doi: 10.22099/mbrc.2024.51170.2037 (PMC11865931; doi:10.22099/mbrc.2024.51170.2037)
Supplement: Supplementary file 1 [file MBRC-14-143-s001.pdf]

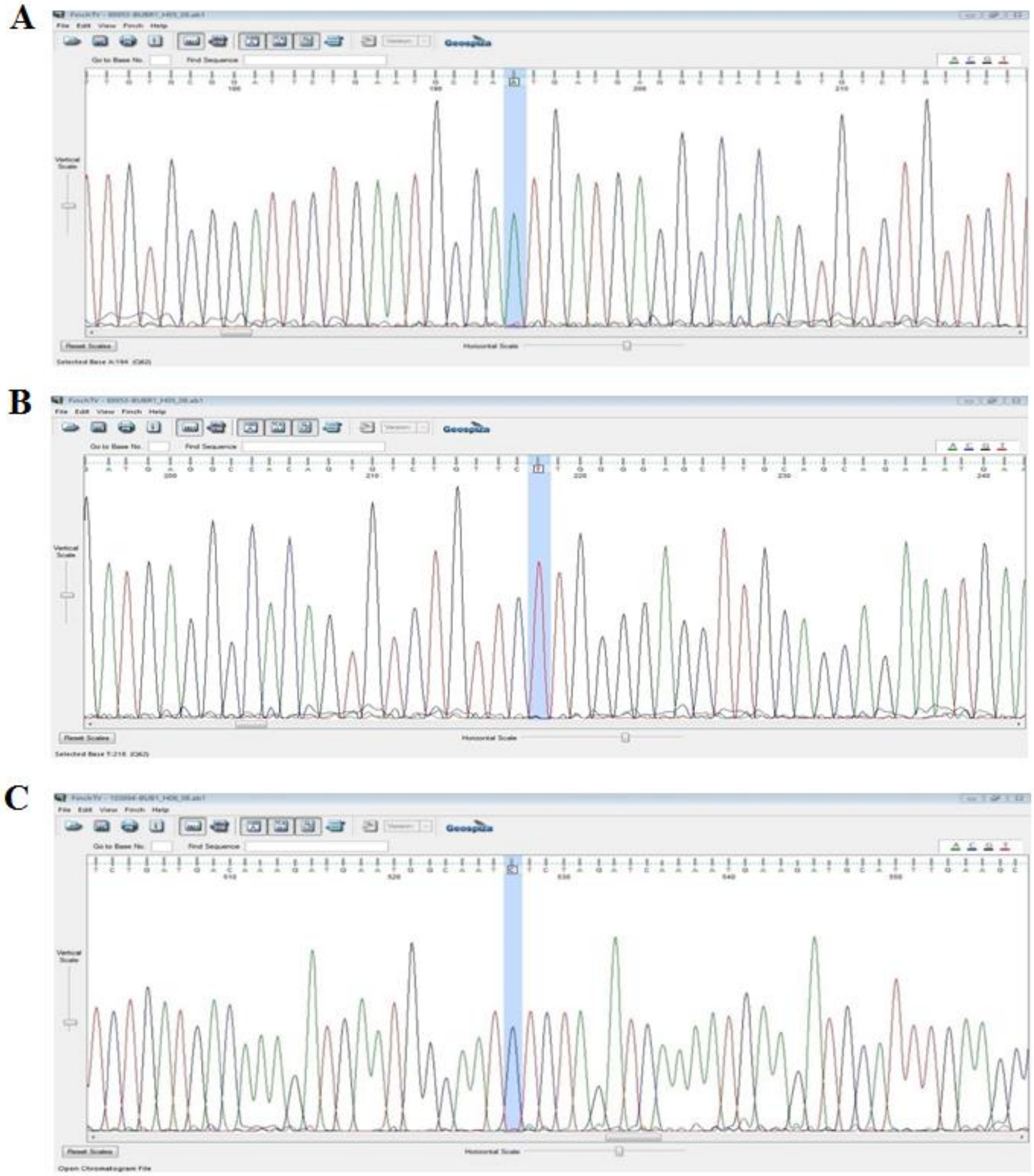

Figure S1: Chromatogram of the variants of interest; the highlights show the locus of variants: a) the rs34998711 on exon23 of *BUBR1*, b) the rs28989185, on exon23 of *BUBR1*, c) the rs121909055 on exon13 of *BUB1*.
